# Molecular aspects of cervical cancer: a pathogenesis update

**DOI:** 10.3389/fonc.2024.1356581

**Published:** 2024-03-19

**Authors:** Verónica Vallejo-Ruiz, Lourdes Gutiérrez-Xicotencatl, Oscar Medina-Contreras, Marcela Lizano

**Affiliations:** ^1^ Centro de Investigación Biomédica de Oriente, Instituto Mexicano del Seguro Social, Puebla, Mexico; ^2^ Centro de Investigación Sobre Enfermedades Infecciosas, Instituto Nacional de Salud Pública, Cuernavaca, Morelos, Mexico; ^3^ Epidemiology, Endocrinology & Nutrition Research Unit, Mexico Children’s Hospital, Mexico City, Mexico; ^4^ Unidad de Investigación Biomédica en Cáncer, Instituto Nacional de Cancerología, Mexico City, Mexico; ^5^ Departamento de Medicina Genómica y Toxicología Ambiental, Instituto de Investigaciones Biomédicas, Universidad Nacional Autónoma de México, Mexico City, Mexico

**Keywords:** cervical cancer, human papillomavirus, E6, E7, oncogenesis

## Abstract

Cervical cancer (CC) is a significant health problem, especially in low-income countries. Functional studies on the human papillomavirus have generated essential advances in the knowledge of CC. However, many unanswered questions remain. This mini-review discusses the latest results on CC pathogenesis, HPV oncogenesis, and molecular changes identified through next-generation technologies. Interestingly, the percentage of samples with HPV genome integrations correlates with the degree of the cervical lesions, suggesting a role in the development of CC. Also, new functions have been described for the viral oncoproteins E5, E6, and E7, resulting in the acquisition and maintenance of cancer hallmarks, including proliferation, immune response evasion, apoptosis, and genomic instability. Remarkably, E5 oncoprotein affects signaling pathways involved in the expression of interferon-induced genes and EGFR-induced proliferation, while E6 and E7 oncoproteins regulate the DNA damage repair and cell cycle continuity pathways. Furthermore, next-generation technologies provide vast amounts of information, increasing our knowledge of changes in the genome, transcriptome, proteome, metabolome, and epigenome in CC. These studies have identified novel molecular traits associated with disease susceptibility, degree of progression, treatment response, and survival as potential biomarkers and therapeutic targets.

## Cervical cancer pathogenesis

Cervical cancer (CC) remains a public health problem and ranks fourth in cancer mortality in women worldwide (1). The main etiologic factor for CC development is a persistent infection with high-risk (HR) human papillomavirus (HPV), responsible for almost 100% of all CC cases (2). However, some studies report that between 5 and 8% of CC cases are HPV-negative; significantly, the majority are adenocarcinomas (3, 4).

More than 200 HPV types have so far been identified. Around 15 types are classified as high-risk types, including HPV 16, 18, 31, 33, 45, 52, and 58, associated with cervical, anogenital, and oropharyngeal cancers, and HPV16 is found in approximately 60% of the CC cases (5). Low-risk HPV types, mainly types 6 and 11, commonly cause benign anogenital warts.

HPVs are small, non-enveloped viruses with an 8-kb circular double-stranded DNA contained in a 55 nm icosahedral capsid. The viral genome holds the long control region (LCR) that regulates genome replication and transcription of the early (E1-E7) and the late-expressed genes L1 and L2 (6).

HPV infection targets the cervix transformation zone, a region susceptible to the development of premalignant cervical lesions and, potentially, cancer. Initially, these lesions were categorized based on their severity and extent of atypical epithelial tissue changes, leading to the classification of cervical intraepithelial neoplasia (CIN) grades I, II, and III, as well as carcinoma *in situ* (CC). Later the Bethesda classification was introduced, revising the terminology. Under this system, CIN I was renamed low-grade squamous intraepithelial lesion (LSIL), whereas CIN II-III were collectively designated as high-grade SIL (HSIL). This updated classification reflects a more refined understanding of the progression and implications of HPV-related cervical lesions (7).

Through micro-wounds, HPV infects the basal cells of the stratified cervical epithelium. A mandatory cellular uptake receptor for HPV has not yet been identified; however, it has been proposed that the virus may attach to the host cell membrane via heparan sulfate proteoglycans (HSPGs) (8), α6β4 integrin complex (9), tetraspanins (10), keratinocyte and epidermal growth factor receptors (KGFR and EGFR, respectively), being EGFR signaling essential for HPV16 endocytosis (11). The virus internalizes by endocytic uptake and, sequentially, in endosomal compartments, the viral capsid binds to retromer components such as Sortin-nexin 17 and 27, helping the L2-DNA complex to escape lysosomal degradation (12, 13) to be then transported to the nucleus via dynein-mediated transport through microtubules (14). Synthesized E1 and E2 interact with the origin of replication site in the LCR. Subsequently, E2 partially represses the expression of E6 and E7; however, the small amount of oncoproteins produced is sufficient to induce a delay in differentiation, resulting in a low copy number of genome replication. Paradoxically, cell differentiation is required to activate the productive phase of the viral cycle. As the epithelium differentiates, the expression of the early viral genes, including E5 and E4, augments in the middle and upper layers, and genome amplification increases (15). E5 maintains cell proliferation and delays cell differentiation by modulating EGF/KGF receptor activities, complementing the functions of E6 and E7 (16). L1 and L2 capsid proteins are produced in the differentiated keratinocytes, where virions are assembled and released due to the disruption of the cytoskeleton promoted by the E4 (17). Most HPV infections are eliminated by the immune system, where 60% of HPV infections are cleared spontaneously within one year and 90% within two years (18). HPV infections that persist over two years exhibit an increased risk of developing cervical intraepithelial neoplasia (CIN) (19).

The determinants of HR-HPV persistent infections are not precise. Still, they may be related to the inability of the host to mount an adequate innate immune response and a robust cell-mediated immunity, as well as the ability of the viral proteins to evade immune detection (20–22). Although only 0.6% of HPV infections are known to progress to cancer, a 16-year follow-up study has shown that the risk of developing cancer is 75.4 times higher in women with persistent HR-HPV infections compared to HPV-negative women (23). It is proposed that during persistent HPV infections, mutations and chromosomal abnormalities accumulate over time, promoting integration of the viral genome into the cellular genome and contributing to cancer progression (24). Some studies report that the frequency of HPV genome integration gradually increases as cervical lesions progress, observed in 26-30% of CINI cases, 40-64% of CINII-III, and 77% of CC (25, 26).

In many cases of CC, the viral genome integration frequently occurs in the E1 and E2 genes, affecting their expression and leading to the uncontrolled expression of the oncogenes E6 and E7. The maintenance of the tumor phenotype requires the continuous expression of the E6 and E7 oncogenes (27). It has been shown that in the HPV genome of CC tumors HPV16-positive, gene losses of more than 10% occur most frequently within E1, E2, and E5 genes, with the loss of E2 in 27% of CC cases (28). However, viral integration does not occur in all CC; in some cases, HPV DNA remains as an episome (29), and methylation at E2 binding sites within the LCR has been shown to prevent E2 binding and consequently promote the continued expression of the viral oncoproteins (30).

Gene loss, duplication, or overexpression is a common feature of the HPV genome in CC (31). This is due to deletions, errors during the replication process, or mutations in genes of the HPV genome that increase the expression of the HPV gene products. HPV gene diversity and duplication have been reported in CC (32). Overexpression of E6 and E7 after HPV integration is considered the trigger for malignant progression due to cell cycle disruption and induction of genome instability; also, multiple HPV integration events have been associated with poor prognosis (31). Alterations that occur in the host genome due to the integration of HPV are fundamental in the development of CC.

## HPV-associated cervical cancer oncogenesis

The contribution of HPV to CC development is due to the transformation capacity of a variety of interactions of E6, E7, and E5 viral oncoproteins with diverse cellular proteins, which affect the normal regulation of cell signaling pathways involved in proliferation, DNA damage repair, immune system, apoptosis, and metabolism (33–35).

The viral oncoprotein E5 is a viroporine capable of increasing cell proliferation by modulating ionic homeostasis through the inhibition of the vacuolar H+ ATPase-16 kDa subunit (36). E5 regulates endosomal pH and inhibits the interaction of EGFR with c-Cbl ubiquitinase, decreasing its degradation and allowing increased mitogenic signal transduction (37). E5 negatively modulates the CDK inhibitors p27Kip-1 and p21Waf-1 (16), which allows the cell to remain in the cell cycle, maintaining viral persistence. E5 also impairs keratinocyte differentiation by downregulating KGFR and increases EGFR activity, slowing the differentiation process (38). All these events lead the cell to a continuous proliferation that could finally transform the cells (**Table 1**).

**Table 1:** Target proteins of HPV oncogenes.

HPV Oncoprotein	Target protein	Related Activity	Reference
E5	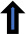 EGFR	Up-regulated mitogenic activity	(37)
	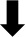 Cbl	Modulation of EGFR degradation by ubiquitination	(37)
	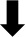 p21^Waf-1^, p27^Kip-1^	Down-regulated inhibitors to remain in the cell cycle	(16)
	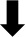 KGFR	Regulation of cell differentiation	(38)
	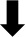 H^+^ ATPase-16 kDa	Modulation of endosomal pH	(36)
	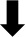 MHC-I and MHC-II	Evasion of immune response	(39, 40)
E6	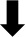 TNF	Apoptosis modulation	(41)
	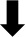 p53	Inhibition of apoptosis	(42)
	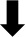 IRF3	Disruption of INF-β signaling	(43, 44)
	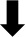 TLR9	Evasion of immune response	(45)
	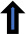 Telomerase	Inhibition of senescence	(46)
	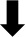 Proteins with PDZ domains	Proteolytic degradation of different target proteins (hDlg, hSCRIB, MAGI-1, -2, -3, NHERF-1)	(47, 48)
	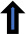 Wnt/β-catenin pathway	Enhanced transcription of growth and proliferation genes	(49)
E7	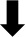 pRb, p107, p130, p600	Release of cell cycle	(50, 51)
	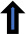 Cullin 2	Polyubiquitination of pRb	(52)
	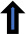 E2F1	Increased transcription of cell cycle genes	(51, 52)
	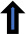 CDK2/Histone H1 kinase	Increase viral DNA replication and cell cycle G1/S checkpoint inhibition	(53)
	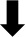 Hijacks RNF168 E3 ubiquitin ligase	Reshape of DNA damage response	(54)
	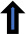 PI3K/AKT/SGK	Promotion of cell cycle and cell survival involving NCAPH function	(55)
	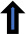 HIF-1α	Increase HIF-1 transcription activity	(56)
External cofactors			
Hypoxia	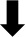 E6/E7	Evasion of senescence	(57)
Microbiome	* 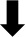 Lactobacillus* spp	Alteration of cervical microenvironment	(58, 59)

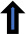
Overexpression and 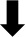
Subexpression induced by the viral oncogenes or external factors.

Additionally, E5 oncoprotein interferes with the host’s immune system, promoting HPV persistence and resistance to immunotherapy; this has been demonstrated through E5 interaction with the simulator of interferon genes (STING), which suppresses interferon (IFN) signaling pathway (60). E5 obstructs the immune response by retaining MHC-I molecules in the ER and Golgi, reducing cell surface antigen presentation, and preventing viral antigen recognition and T and NK cell maturation (39). E5 also affects antigen presentation through MHC-II by preventing invariant chain degradation by inducing endosome alkalinization, thus reducing the activity of this molecule on the cell surface (39, 40). Interestingly, HPV16 E5 has been shown to suppress the expression of keratinocytes-specific IFN with the subsequent inactivation of the JAK/STAT pathway and the suppression of IFN-stimulated genes, which finally impacts the maintenance and integrity of the viral episomes (61).

One of the main targets of E6 is the tumor suppressor p53, promoting its degradation through the interaction with the ubiquitin ligase E6AP (42, 62), which induces cells to enter S-phase without arresting in G1. Moreover, E6 can stimulate the transcriptional factor OCT-4 expression, which binds to the p53 promoter recruiting the NCOR1 co-repressor, representing a new mechanism by which E6 decreases p53 levels (63). E6 has anti-apoptotic activities, which may occur by the degradation of p53, leading to the down-regulation of the proapoptotic genes PUMA and Bax and genes related to senescence modulation (64, 65). Moreover, E6 and E7 activate the transcription of Survivin and the apoptosis inhibitor c-IAP2, conferring resistance to apoptosis (66). E6 also prevents apoptotic signals through direct interaction with interferon regulatory factor 3 (IRF3), inhibiting the IFN-β1 response (43), and through hypermethylation of the promoter of the tumor suppressor death-associated protein kinase 1 (DAPK1) leading to the down-regulation of IFN genes, which consequently impairs the cellular antiviral response (44).

E6 sensitizes to radiation therapy by hijacking cellular target proteins involved in DNA damage repair, such as CHEK2, CLK2/3, ERCC3 MNAT1, PER1, RMI1, RPA1, UVSSA, and XRCC6, which promotes their colocalization in HPV replication foci, facilitating viral replication and increasing cellular genome instability (67). Additionally, E6 increases telomerase activity through the degradation of the telomerase inhibitor NFX1-91 and, in conjunction with Myc, transactivates the hTERT catalytic subunit, allowing cell immortalization due to the inhibition of senescence (46).

HR-HPV E6 proteins contain a PDZ-binding motif through which they bind proteins containing PDZ domains, sending them to degradation by ubiquitination, such as hDLG, hSCRIB, MAGI-1, -2, -3, and NHERF-1. In this condition, MAGI-2 cannot interact with PTEN to suppress AKT activation, while degradation of NHERF-1 activates the PI3K signaling pathway, thus promoting cell survival and proliferation (47, 48). In addition, degradation of NHERF1 by the E6/E6AP complex activates the Wnt/β-catenin signaling pathway, leading to the accumulation of β-catenin that induces transcription of genes that regulate cell growth and proliferation (e.g., c-Jun, c-myc, cyclin D, survivin, COX-2) (49).

Recent studies using the mouse papillomavirus (MmPV1) model demonstrated that E6 oncoprotein modulates the Notch signaling pathway by interacting with MAML1, component of the Notch pathway, affecting cell density and delaying differentiation, which allows viral persistence (68).

The E7 oncoprotein promotes cell cycle progression by sequestering and degrading the tumor suppressor protein pRB via polyubiquitination by cullin 2 (CUL2) (52), which releases the E2F transcription factor from the pRb complex, allowing the cell cycle to progress to the S-phase (50, 51). Another player in the pRb-CUL2 degradation pathway is miR-154-5p, which targets CUL2 and is down-regulated by E7 (52). Also, HR-HPV E7 oncoprotein can trigger tumorigenesis in a pRB-independent pathway by binding directly to the E2F1 transcription factor (50) and overexpressing eIF4E translation factor (69), promoting cell proliferation and migration while inhibiting apoptosis. Furthermore, E7 also modulates the G2/M cell cycle phase by upregulating the kinase activity of the histone H1 through the Cyclin A/CDK2/p107/E7 complex, which promotes HPV replication but utilizes ATM signaling to activate the p38/MK2 pathway also required for viral replication (52, 70). Moreover, E7 binds the RN1698 E3 ubiquitin ligase, hijacking its activity to promote viral replication; consequently, cellular response to DNA damage is reshaped, promoting genome instability (54). Interestingly, E7, through E2F1, promotes the expression of the NCAPH gene, which is involved in the activation of PI3K/AKT/SGK signaling and has also been implicated in proliferation, migration, invasion, epithelial-mesenchymal transition and restricts tumor formation (55). Recent findings reveal a complex interplay between PI3K/AKT/mTOR in virus-host cell communication. E6/E7 inhibits cell senescence under normoxia. Still, oxygen deprivation leads to the impairment of mTOR signaling, and hypoxic HPV-positive cells can evade senescence, although E6/E7 is down-regulated due to the activation of AKT (57).

External cofactors are also crucial for HPV-induced carcinogenesis, as is the case of the microbiome. Alterations in the cervicovaginal microbiome occur during the progression of HPV-associated lesions to CC, where an increase in resident bacteria diversity occurs along with a reduction of the resident *Lactobacillus* spp. Dysbiosis in the cervical microbiome influences viral persistence and is a carcinogenic co-factor (58, 59).

**Table 1** shows several cellular targets of viral oncoproteins affecting biological processes.

## New advances in molecular characterization of cervical cancer

The molecular changes in CC are not yet fully elucidated, and next-generation technologies have generated a large amount of information that has increased knowledge in this area. Bioinformatic data analysis has provided relevant information on the genome sequence, transcriptome, proteome, glycome, epigenome, etc. Several studies have revealed molecular changes that occur during the development and progression of CC and have identified potential biomarkers and molecular targets associated with susceptibility to the disease, degree of advancement, response to treatment, and survival.

CC depends largely on the genetic characteristics of the host. Koel et al. (2023) (71) performed a genome-wide association study (GWAS) to identify genomic variants associated with the full spectrum of cervical disorders, including ectropion, cervicitis, dysplasia, and CC. The variants that were mainly identified overlapped between cervicitis, dysplasia, and cancer and appeared different for ectropion. A genetic risk score (GRS) associated with CC was constructed. GRS identified people at risk of developing CC, which could be used to personalize the screening strategies for susceptible people. Most of the predictive power of the variants identified comes from the HLA region (HLA-DQA1), but it also includes regions where the closest genes are CDC42, PAX8, CLPTM1L, and ORMDL3.

During HPV carcinogenesis, somatic mutations are accumulated in the host genome. Through the whole exon-, genome- and transcriptome sequencing, driver mutations have been identified in the genomic landscape of an important number of CC samples paired with normal cervical tissue (72). Among the most frequent alterations found in CC were somatic mutations in *MAPK1* (8%), *EP300* (16%), *FBXW7* (15%), *PIK3CA* (14%), *PTEN* (6%), *NFE2L2* (4%), *TP53* (5%), *ERBB2* (6%) and *HLA-B* inactivating mutations (9%). Furthermore, in cases of cervical adenocarcinoma, the most frequent somatic mutations were found in *ELF3* (13%) and *CBFB* (8%). Moreover, Zhou L. et al. (2022) (31) characterized virus-human integration events in CC samples, combined with the corresponding RNA-seq analysis. Among the genes frequently found disrupted by HPV integration were *IL20RB, SOX14, LENG8, LENG9, CDC42EP5, CASC21, CCAT2, CASC8*, and *AKAP13*, while genes whose expression was altered included *LINC00290, LENG9, CCAT2, ARHGAP42, HNF1B, FOXD2, TMC1, IL15*, and *RPS6KA.*

Transcriptome analyses in CC have identified changes in the expression of genes involved in cell transformation and immune response. Moreover, other RNAs involved in the disease have been identified, such as miRNAs, circRNAs, and long ncRNAs, that play essential roles in cancer pathogenesis as tumor promoters or suppressors (73–75). Salmerón-Bárcenas et al. (2023) (76) performed a bioinformatic analysis focusing on miR-182-3p, which is described in other cancers associated with chemoresistance and cancer progression. They identified miR-182-3p gene targets downregulated in CC and found that it might participate in angiogenesis and cell migration. They also proposed this miR as a potential diagnostic biomarker.

Understanding of the biological roles of circRNAs in CC progression is still under investigation, and the mechanisms by which circRNAs influence CC development and metastasis have yet to be completely elucidated. Zhang et al. (2023) (77) performed an expression analysis using microarray technology and identified differentially expressed mRNAs, miRNAs, and circRNAs in CC tissues. Their study focused on analyzing circRNAs with binding sites for miR-154-5p, a tumor suppressor in CC. hsa_circ_0000276 had the most substantial binding capacity for miR-154-5p and showed an increased expression in CC tissue. Through bioinformatic analysis, the authors showed that hsa_circ_0000276 was associated with CD47, LDHA, PDIA3, and SLC16A1, related to immune system processes; it was also reported that hsa_circ_0000276 increased proliferation and inhibited apoptosis.

Through lncRNA/mRNA microarray technology, Xin et al. (2023) (78) identified differentially expressed lncRNAs and mRNAs in HPV16 and HPV18-positive CC tissues compared with normal tissues. They found that co-expression of LINC00511-PGK1 could be important in the HPV-mediated transformation. Then, a score was proposed based on the co-expression of LINC00511 and PGK1 that predicted the overall survival of the patients with CC.

Other studies have focused on analyzing genes involved in ubiquitination, whose role in CC has yet to be determined. Hao et al. (2023) (79) identified the ubiquitination-related genes differentially expressed in CC tumors. They selected those associated with overall survival and established a prognostic gene signature that includes *RBBP4, SRM, GCH1, USP14, TRAIP, CBX4, VEZF1*, and *TOM*1 genes. Considering the importance of the anti-tumor immune response to control cancer development and progression, other groups have reported immune gene signatures. Pu et al. (2022) (80) reported an immune signature composed of 10 genes: *CD96, LAG3, PDCD1, TIGIT, CD27, KLRK1, LTA, PVR, TNFRSF13C*, and *TNFRSF17*; these genes are CD79B associated immunomodulators, that showed an independent prognostic value. A study that analyzed RNAseq databases of genes related to the glycosylation process (glycogens) showed that adenocarcinoma tumors displayed a unique glycogen expression signature. Squamous cancers showed more significant heterogeneity since six different signatures were identified related to different glycosylation pathways, such as glycosphingolipids, keratan and heparan sulfate synthesis, and glycosaminoglycan degradation (81).

Epigenetic changes have been evaluated in CC. Analyzing the Cancer Genome Atlas DNA methylation database, Yang et al, 2020 (82), identified HPV-related methylation sites in the DNA of CC tumors and classified them into clusters associated with overall survival. Aberrant mutations, amplifications, and deletions were identified in the different methylation groups, proposing a prognostic signature that allows patients to be stratified into high and low risk. Salta et al. (2023) (83) performed a meta-analysis on DNA methylation in HR-HPV-positive women with HSILs and proposed DNA methylation-based markers to discriminate lesions with a higher risk of progression to CC.

## Conclusions

Current research on HPV has primarily focused on E6 and E7 oncogenes due to their impact on the development of CC. These genes are often found overexpressed in CC cells. Some promising approaches targeting E6 and E7 include the development of therapeutic vaccines for preventing the progress of squamous cell intraepithelial lesions to CC (84), antibodies against viral oncogenes to inhibit tumor growth (85, 86), strategies that inhibit their expression (87, 88), and immunotherapy that uses the host’s immune system to attack HPV-related cancers (89). Furthermore, E5 has gained relevance for participating in the progression and maintenance of CC. Moreover, novel bioinformatic analyses have identified vital genes, miRNAs, lncRNAs, circRNA, and signaling pathways contributing to CC progression. Some of these findings have been proposed not only as potential biomarkers but also as therapeutic targets. As research on CC continues, our knowledge of the genetic changes contributing to this disease increases, which could eventually help improve diagnostic tests and treatment options for this type of cancer.

## Author contributions

VV-R: Writing – review & editing, Writing – original draft, Supervision, Investigation, Conceptualization. LG-X: Writing – review & editing, Writing – original draft, Investigation, Conceptualization. OM-C: Writing – review & editing, Writing – original draft, Investigation, Conceptualization. ML: Writing – review & editing, Writing – original draft, Supervision, Investigation, Conceptualization.
